# Hypertriglyceridemic Waist Phenotype and Its Association with Metabolic Syndrome Components, among Greek Children with Excess Body Weight

**DOI:** 10.3390/metabo13020230

**Published:** 2023-02-03

**Authors:** Eirini Dikaiakou, Fani Athanasouli, Anatoli Fotiadou, Maria Kafetzi, Stefanos Fakiolas, Stefanos Michalacos, Elpis Athina Vlachopapadopoulou

**Affiliations:** 1 Department of Endocrinology-Growth and Development, Children’s Hospital P. & A. Kyriakou, 11527 Athens, Greece; 2 2nd Department of Paediatrics, “P&A Kyriakou” Children’s Hospital, University of Athens, 11527 Athens, Greece; 3 Biochemistry Department-Hormones Laboratory, Children’s Hospital P. & A. Kyriakou, 11527 Athens, Greece

**Keywords:** obesity, children, hypertriglyceridemic waist phenotype, insulin resistance, HDL, HOMA-IR, triglycerides

## Abstract

The hypertriglyceridemic waist (HTGW) phenotype is characterized by abdominal obesity and elevated serum triglycerides. We aimed to assess the prevalence of the HTGW phenotype among children with overweight or obesity and its association with indices of insulin resistance (IR) and dyslipidemia. A total of 145 children with mean age of 10.2 years (SD = 2.31 years), 97.2% of whom with obesity, were analyzed. The HTGW phenotype was defined as WC > 90th Centers for Disease Control and Prevention (CDC) percentile and triglyceride levels of ≥100 mg/dL and ≥130 mg/dL for children 0 to 9 or >10 years of age, respectively. In total, 77.9% of the children had a waist circumference above the 90th percentile and 22.8% had elevated triglycerides. The prevalence of the HTGW phenotype in this sample was 19.3%. Patients with the HTGW phenotype had significantly lower levels of High-Density Lipoprotein (*p* < 0.001) and were insulin-resistant, as evident by an increased mean Triglycerides Glucose Index 8.64 (SD = 0.24) vs. 7.92 (SD = 0.41) for those without the HTGW phenotype (*p* < 0.001), and increased prevalence (54.5%) of Homeostasis Model Assessment of Insulin Resistance (HOMA-IR) in ≥2.5 in patients with HTGW (*p* = 0.045). Children with the HTGW phenotype were more likely to have increased HOMA-IR [OR 7.9 95% CI (1.94, 32.1)]. The HTGW phenotype is a low-cost and easily available index that might help to identify children with increased cardiometabolic risk.

## 1. Introduction

Obesity is recognized as a chronic disease in childhood and adolescence, affecting a rising number of children and leading to metabolic and cardiovascular comorbidities as well as long-term complications [1,2]. According to the World Health Organization (WHO), one-third of children in the WHO European region are diagnosed as being overweight or having obesity. In the Greek population, the prevalence of pediatric abdominal obesity is reported to be among the highest worldwide, and this is very concerning, as abdominal obesity is recognized as a significant predictor of cardiovascular risk [3]. Furthermore, 60% of children who are overweight before puberty will retain their overweight status as young adults; in almost all developing or developed countries, the prevalence of obesity rises among children and adolescents aged 5–19 years. Thus, it is considered as one of the most serious public health issues that threatens future health and longevity [4].

Metabolic syndrome (MetS) is a constellation of metabolic abnormalities, such as central obesity, glucose intolerance, dyslipidemia and hypertension. Despite extensive research, there is no consensus for the definition and the diagnostic criteria, thus diagnosis in the pediatric population remains controversial [5]. However, clinical and metabolic alterations in MetS are linked to increased cardiometabolic risk [6]. There are various clinical and laboratory tools proposed to identify people at increased cardiometabolic risk, associated with abdominal obesity and central adiposity. Body mass index (BMI) is a simple index of weight-for-height, commonly used to classify overweight and obesity; however, waist circumference (WC) is an anthropometric measurement that shows a stronger correlation with intra-abdominal fat than BMI [1].

The hypertriglyceridemic waist (HTGW) phenotype is characterized by the simultaneous presence of enlarged WC and hypertriglyceridemia [7]. Lemieux et al. suggested the presence of an atherogenic metabolic triad for HTGW phenotype: increased serum apolipoprotein B concentrations, high serum concentrations of small dense low-density lipoprotein (LDL) and insulin resistance (IR) [7]. The HTGW phenotype is an index that can discriminate subcutaneous from visceral obesity and predict disorders, such as cardiovascular disease (CVD), metabolic syndrome (MetS) and type 2 diabetes mellitus (T2DM) [7,8]. Furthermore, HTGW has been linked to insulin resistance and the subsequent development of T2DM in adults [9].

Since the introduction of the HTGW phenotype concept, there is growing interest in investigating its correlation with different components of MetS. There are numerous studies concerning the HTGW phenotype conducted in the adult population. The HTGW phenotype in adults is associated with the presence of CVD and may be an alternative to MetS to detect those at risk [10,11,12]. In a cross-sectional study in China, hypertensive adults with the phenotype had a higher prevalence of hypercholesterolemia, high LDL, low HDL and hyperuricemia, and thus an increased cardiometabolic risk [13]. Moreover, the HTGW phenotype has been correlated with prediabetes and diabetes [14,15,16,17,18], as well as with atherogenic and coronary artery disease [17,18,19]. Yu D et al., reported a link between HTGW phenotype and abnormal hepatic and renal function (higher concentrations of alanine aminotransferase (ALT) and a reduced eGFR) [20].

There is significant evidence in the literature associating HTGW phenotype among adults and metabolic risk factors; however, there are limited data concerning the prevalence of HTGW phenotype in children and adolescents and its association with metabolic comorbidities [21,22]. Bailey et al., demonstrated that the HTGW phenotype is associated with cardiometabolic risk in children and adolescents and is a more sensitive marker than waist to height ratio (WHR) for identifying subjects at risk [23].

This study aims to assess the prevalence of HTGW phenotype among children with overweight and obesity followed at a referral center in Greece and further investigate whether there is an association between HTGW phenotype and other metabolic risk factors, such as IR, hypertension, and suspected fatty liver (NAFLD). To our knowledge, there are no data regarding the prevalence of HTGW phenotype in Greek pediatric population.

## 2. Materials and Methods

### 2.1. Study Design and Population

The study included 145 children (68 males), who were investigated, in the Department of Endocrinology, Growth and Development, “P. & A. Kyriakou” Children’s Hospital, Athens, Greece between 2013 and 2016. A total of 32.4% of the participants had entered puberty according to the Tanner staging. All the patients were referred for investigation and treatment of increased body weight. Children were excluded from the analysis, if they had obesity related syndromes (such as Prader–Willi, Bardet–Biedl, Down, Alström, Laron, DiGeorge and other syndromes) [24], type 1 (T1DM) or type 2 (T2DM) diabetes, chronic kidney or CVD, long-term corticosteroid use, primary hyperlipidemia, as well as any other reason for hepatic steatosis, such as medication (amiodarone, L-asparaginase, valproic acid), cystic fibrosis, HIV, hepatitis B or C, Wilson’s disease or celiac disease.

The investigations were carried out under their routine care and approval was given for the retrospective analysis of the medical record data by the Ethics Committee of the “P. & A. Kyriakou” Children’s Hospital.

### 2.2. Anthropometric Measurements and Blood Pressure

Participants’ height, weight, WC, systolic arterial pressure (SBP) and diastolic arterial pressure (DBP) were measured. Weight and height were measured by well-trained personnel while subjects were in light clothing and barefoot. Participants’ height was measured using a wall-mounted Harpenden Stadiometer Holtain Ltd. Their weight was measured with an electronic scale (SOEHNLE Professional 2755) to the nearest 0.1 kg. WC was measured twice, midway between the lowest border of rib cage and the upper border of iliac crest with the use of inextensible anthropometric tape while the child was standing with their arms at their sides and feet closed together [22]. All measurements were taken twice, and the two measurements were averaged for analysis.

SBP and DBP values were the mean of three measurements after a 5-min rest, with a 1-min interval between each measurement, and they were measured with a calibrated G-Care SP-800 sphygmomanometer. A pediatric cuff of proper size was chosen, so that its bladder width was at least 40% of the arm circumference midway between the olecranon and the acromion, and it covered 80 to 100% of the circumference of the arm [25]. BMI was calculated as weight (in kilograms) divided by height (in meters) squared.

### 2.3. Assays

After a 12-h fast, glucose, lipid profile, aspartate aminotransferase (AST), ALT and insulin levels were measured.

Venous samples were collected in WEGO serum vacuum tubes with a clot activator but without any other additives. Samples were centrifuged for 10 min at 3000 rpm, at room temperature (RT) except for the insulin samples that centrifuged at 20 °C. Serum glucose levels, fasting serum lipids [serum triglycerides (Tg), total cholesterol (TC), high-density lipoprotein (HDL) cholesterol, low-density lipoprotein (LDL) cholesterol], AST and ALT were measured as soon as possible, but not more than 45 min after the blood was drawn by enzymatic, colorimetric methods in a Cobas c501 chemistry analyzer (Roche Diagnostics).

The intra- and inter-assay coefficient variability (CV) of glucose measurement in our laboratory was less than 1.7%.

The intra- and inter-assay coefficient variability (CV) of triglycerides measurement in our laboratory is less than 1.4%. Fasting insulin was measured using the immunometric reaction, ECLIA (electrochemiluminescence method) Elecsys 2010, Roche Diagnostics, Greece, all conducted in a CLIA (clinical laboratory improvement amendments) approved laboratory.

### 2.4. Definitions

Childhood obesity was defined as having a BMI equal to or greater than the sex- and age-specific 95th percentile of the Centers for Disease Control and Prevention (CDC) anthropometric reference data for children and adults, 2007–2010 [1]. Furthermore, a child or adolescent ≥2 years of age was considered as overweight if their BMI was ≥85th percentile but <95th percentile [1]. Abdominal obesity was defined as WHR ≥0.5 and WC equal to or greater than the sex- and age-specific 90th percentile [26]. Triglycerides Glucose Index (TyG) and Homeostasis Model Assessment of Insulin Resistance (HOMA-IR) indices were used as predictors of insulin resistance [27,28]. The TyG index was calculated as the Ln [fasting triglycerides (mg/dL) × fasting glucose (mg/dL)/2] [27]. The HOMA-IR index was calculated as fasting plasma insulin (FPI U/l) × fasting plasma glucose (FPG mg/dL)/405 [28]. A cut-off value ≥2.5 was used for HOMA-IR [29,30,31]. Elevated arterial pressure was defined as SBP or DBP ≥90th percentile [32]. ALT ≥25.8 U/L (boys) and 22.1 U/L (girls) was defined as abnormal in this study, indicating a suspected fatty liver [33]. The HTGW phenotype was defined as WC >90th CDC percentile and triglyceride levels ≥100 mg/dL for children 0 to 9 years of age and ≥130 mg/dL for 10 to 19 years old [1]. MetS was defined according to criteria by Cook et al. [34] in the presence of at least three of the following variables: increased WC for gender and age (≥90%), increased BP for gender, age and height (≥90%), FPG ≥ 100 mg/dL, HDL ≤40 mg/dL, triglycerides ≥110 mg/dL. Dyslipidemia was defined as TC ≥ 200 mg/dL and high LDL cholesterol as ≥130 mg/dL. The midpoint value for HDL cholesterol (<40 mg/dL) was used as a 10th percentile value [35].

### 2.5. Statistical Analysis

All continuous variables followed normal distribution and are expressed as mean values with standard deviation (SD), while categorical variables are expressed as absolute values and percentages in parentheses. Student’s *t*-tests were used for the comparison of means and Chi-square tests for the comparison of proportions. To further evaluate the association of the HTGW phenotype with insulin resistance, we stratified children included in the analysis as follows: (1) those with a normal WC and triglycerides level, (2) those with one component of the HTGW phenotype (either high WC or high triglycerides) and (3) those with the HTGW phenotype. Univariate logistic regression analyses were used to produce odds ratios with 95% confidence intervals for the association of insulin resistance (HOMA-IR ≥2.5) with the HTGW phenotype as well as other anthropometric and metabolic characteristics. The variables that had significant association in univariate logistic regression models were used in multivariable model to determine the independent association with HOMA-IR. Sex was used in multivariable model regardless as a possible confounding. The same logistic regression analyses were used to assess the association of MetS with the HTGW phenotype. Statistical significance was defined as *p* value < 0.05. Statistical analyses were performed using STATA V13.1 (Stata Corp., College Station, TX, USA).

## 3. Results

Demographics, anthropometric and biochemical parameters of the total sample are presented in Table 1. One hundred and forty-five children (46.9% boys) with a mean age of 10.2 years (SD = 2.31 years) were analyzed. Ninety-seven point two per cent (97.2%) had obesity and the rest of them were overweight. Fifty-four point five per cent (54.5) had insulin resistance, according to the HOMA-IR index. Ninety-nine per cent (99.3%) of the participants had WHR ≥ 0.5. Seventy-seven point nine per cent (77.9%) of the participants had a WC above the 90th percentile and 22.8% had elevated triglycerides. The HTGW phenotype was present at 19.3% of the sample.

As shown in Table 2, no gender or age differences were found among children with or without the HTGW phenotype. Similarly, SBP and DBP as well as WHR ≥ 0.5 did not differ among subjects with or without the presence of the HTGW phenotype. Significantly lower levels of HDL were found in cases with the HTGW phenotype compared with those without (*p* < 0.001). However, TC and LDL did not differ statistically significantly in the presence of the HTGW phenotype. Furthermore, among participants with the HTGW phenotype, 71.4% had HOMA-IR ≥ 2.5 vs. 50.4% of participants without the HTGW phenotype, which was statistically significant (*p* = 0.045). Elevated ALT was not found to be associated with the presence of HTGW (*p* = 0.324).

Finally, the mean TyG was significantly higher for those with HTGW compared to those without HTGW (8.79 ± 0.32 vs. 8.0 ± 0.37, *p* < 0.001).

In univariate logistic regression models, age, puberty, HTGW phenotype, SBP and HDL were significantly associated with HOMA in children with obesity. In the multivariable model, after adjustment for these confounders, the odds for insulin resistance were increased with each added HTGW component (Table 3). Children with obesity and the HTGW phenotype were 7.9 times more likely to have HOMA-IR ≥ 2.5 compared with children with obesity and normal WC and triglycerides levels.

Regarding MetS, 21 (75%) patients with the HTGW phenotype met the criteria of MetS. In univariate logistic regression analyses, the HTGW phenotype and HDL were significantly associated with MetS, as was expected, while there was a trend of association with pubertal status and total cholesterol. In multivariable analysis, after adjustment for age, sex, puberty status and total cholesterol levels, the HTGW phenotype and HDL levels remained significantly associated with MetS (Table 4).

## 4. Discussion

To our knowledge, this study represents the first study in the Greek pediatric population that assessed the prevalence of the HTGW phenotype and its correlation with metabolic risk factors. In the current study, the HTGW phenotype was present in 19.3% of Greek children and adolescents with overweight or obesity. No statistically significant gender or age differences were found among children with HTGW compared to those without the phenotype. There are limited data regarding the HTGW phenotype in the pediatric population. The reported prevalence of the HTGW phenotype in adolescents varies widely between 3.3% in China, 6.4% in Iran and 7.3% in the UK [23,36,37]. A study conducted in Brazil, which estimated the HTGW phenotype in students of both genders in public and private schools, found that its prevalence was in 20.7% of the total cases. Fourteen point one per cent (14.1%) were identified in males and 6.6% among females [21]. The great prevalence variability among the different study groups can be attributed to the different cut-off points used to assess WC. In the scientific literature, the cut-off points for the WC range were from the 70th to the 90th percentile for age and gender [23,38]. Furthermore, there is no consensus for the triglycerides’ normal range cut-off values [23,38,39]. Moreover, studies were conducted among populations of different ethnicities.

According to the present data, the HTGW phenotype was associated with insulin resistance and lower HDL levels, a combination consistent with atherogenic tendencies. Similar findings were reported for children of different ethnic backgrounds. Alavian et al. investigated 4811 Iranian school students and demonstrated that children with this phenotype were more likely to have cardiovascular risk factors, notably the overweight ones and/or those with hypercholesterolemia [38]. Esmaillzadeh et al. reported that Iranian adolescents with the HTGW phenotype were more likely to have low HDL, high LDL, hypercholesterolemia and elevated blood pressure (BP) [39]. The study of Conceição-Machado et al. also indicated the significantly positive correlation between the HTGW phenotype and low HDL-C [40].

Visceral or intra-abdominal adiposity is the most usual cause of insulin resistance and has been identified as an important predisposing factor for the development of T2DM. In this study population, the HTGW phenotype was associated with two different insulin resistance indices, the HOΜA-IR and Tyg index. Subjects with the HTGW phenotype had a greater proportion of HOMA-IR ≥ 2.5. Children with obesity and high WC or triglycerides levels had 4.27 greater odds to have HOMA-IR ≥ 2.5, whereas those with HTGW phenotype had 7.9 greater odds to have HOMA-IR ≥ 2.5 compared with those with normal WC and triglycerides levels. Moreover, the mean TyG was 8.64 (SD = 0.24) for those with the HTGW phenotype and 7.92 (SD = 0.41) for those without HTGW phenotype (*p* < 0.001). Limited information is available on the effect of the HTGW phenotype on insulin resistance in the pediatric population. BRAMS study indicated a strong correlation between the HTGW phenotype and IR and metabolic syndromes in Brazilian adolescents [41]. The study of Buchan et al. also demonstrated that children with the HTGW phenotype had significantly higher cardiometabolic risk scores (based on four variables: SBP, TC:HDL-c ratio, HOMA-IR and CRP) when compared with children without the phenotype [42].

WHR is a measure of abdominal obesity and body fat distribution and appears to be a strong predictor of cardiovascular risk factors among children, independently of gender and ethnic groups [43]. The research on the relationship between WHR and the HTGW phenotype in the pediatric population is very limited. Liu et al. conducted a population-based study to investigate the hypothesis that WHR can detect adolescents at increased risk of the HTGW phenotype. They demonstrated a high prevalent HTGW phenotype in subjects with increased WHR [37]. In our study, 99.3% of the participants had WHR ≥ 0.5, as was expected due to our study sample. Therefore, WHR ≥ 0.5 did not differ significantly among subjects with or without the presence of the HTGW phenotype and it could not be used to discriminate the two groups.

Regarding the elevated ALT in the presence of the HTGW phenotype, we did not find any statistically significant positive association between NAFLD risk and the HTGW phenotype. Additionally, we did not prove any significant association between the HTGW phenotype and elevated BP. However, other studies in adolescents, such as those of Esmaillzadeh and Ribero, have demonstrated that the HTGW phenotype was positively associated with higher levels of BP [39,41].

Finally, according to our findings, children with obesity and HTGW phenotype are more likely to have MetS. In our study, 75% of children with the HTGW phenotype met the criteria of MetS. There are limited data regarding MetS in the Greek pediatric population. Vlachopapadopoulou et al. reported a high prevalence of MetS (12.7%) in a cohort of one hundred eighty-nine Greek pre-pubertal children with obesity. Moreover, increased WC, BMI, WHR and acanthosis nigricans were recognized as early clinical indicators for increased metabolic risk [44]. Papandreou et al. investigated one hundred and twenty-five subjects with obesity, aged 11–12 years, for NAFLD risk. Forty-four children (58.6%) were reported to have MetS, while children with MetS and obesity had three times the higher risk of developing NAFLD [45]. To our knowledge, our study is the first to be conducted in the Greek pediatric population, providing data for the association of the HTGW phenotype with MetS, IR, lipid profile, ALT and arterial pressure among children with excess body weight. The analysis of the factors associated with metabolic risk and insulin resistance is essential to be analyzed in pediatric patients of different ethnic backgrounds in order to appreciate biologic variability.

There are limitations in this study that should be considered. The major limitation of this study is that all the participants included in this cohort originate from only one referral center. A larger number of participants would reflect more appropriately the general population. Fat distribution, insulin sensitivity and serum lipid concentrations are affected by pubertal status. Insulin resistance is more evident in adolescence and lipid levels tend to be higher. In our study, two-thirds of the participants were pre-adolescents, and the sample of adolescent participants was limited, not allowing us to compare results between the two samples and draw reliable conclusions. Furthermore, the lack of information on other risk factors, such as level of physical activity, family history and dietary habits could be a possible confounder of our results. Moreover, we recognize that the hyperinsulinemic-euglycemic clamp is the gold standard method to assess insulin resistance. However, in order to overcome this limitation, we measured two insulin resistance indices, HOMA-IR, which is a very commonly used and recognized insulin resistance index, as well as the TyG index, which has been recently appreciated. The use of two indices contributes strength to the results.

The lack of standardization of available classification and cut-off points for the HTGW phenotype, mainly in the pediatric population, as well as the ethnic differences among populations, which influence cardiometabolic parameters, impedes the comparison between different research data sets, indicating that more research is needed in this field.

## 5. Conclusions

This study provided evidence that high prevalence of the HTGW phenotype was detected among Greek children with excess body weight. The HTGW phenotype was not associated with elevated blood pressure or elevated ALT, but it increased by almost eight times the possibility of insulin resistance. It was also associated with lower HDL levels and a higher likelihood of the presence of metabolic syndrome. As the HTGW index is simple, reproducible and low cost, it is a potentially useful tool for the early identification of children who are susceptible to cardiometabolic risk. However, future research is needed for the validation of national cut-off points among different age groups in order to obtain data with greater reliability and applicability.

## Figures and Tables

**Table 1:** Demographic, anthropometric, clinical and biochemical characteristics of participants.

Characteristics	N = 145
Age [years, mean ± SD]	10.2 ± 2.31
Sex [Boys, n (%)]	68 (46.9)
Puberty [n (%)]	47 (32.4)
BMI [Obese, n (%)]	141 (97.2)
WHR [≥0.5, n (%)]	144 (99.3)
WC ≥ 90th [n (%)]	113 (77.9)
BP ≥ 90th [n (%)]	67 (46.9)
HOMA-IR ≥ 2.5 [n (%)]	79 (54.5)
MetS [n (%)]	27 (18.6)
HTGW phenotype [n (%)]	28 (19.3)

Abbreviations: SD, standard deviation; BMI, body mass index; WHR, waist to height ratio; WC, waist circumference; BP, blood pressure; HOMA-IR, homeostasis model assessment of insulin resistance; MetS, metabolic syndrome; HTGW, hypertriglyceridemic waist.

**Table 2:** Characteristics of clinical and anthropometric parameters of participants by HTGW phenotype.

	HTGW Phenotype		
Characteristics	No	Yes	*p*-Value
Age (years, mean ± SD)	10.2 ± 2.30	9.94 ± 2.57	0.581
Sex [Boys, n (%)]	56 (47.9)	12 (42.9)	0.633
Puberty [n (%)]	37 (31.6)	10 (35.7)	0.678
BMI [Obese, n (%)]	114 (97.4)	27 (96.4)	0.770
WHR [≥0.5, n (%)]	116 (99.2)	28 (100)	0.624
BP ≥ 90th [n (%)]	52 (44.8)	15 (55.6)	0.314
SBP (mmHg, mean ± SD)	112.0 (10.2)	112.4 (9.41)	0.840
DBP (mmHg, mean ± SD)	67.2 (10.2)	68.8 (12.9)	0.497
Chol (mg/dL, mean ± SD)	163.9 ± 29.9	172.6 ± 19.2	0.154
HDL (mg/dL, mean ± SD)	52.5 ± 12.3	43.3 ± 8.81	<0.001
LDL (mg/dL, mean ± SD)	98.6 ± 27.6	102.5 ± 18.3	0.506
HOMA-IR ≥ 2.5 [n (%)]	59 (50.4)	20 (71.4)	0.045
Elevated ALT [n (%)]	34 (32.1)	11 (42.3)	0.324
TyG (mean ± SD)	7.92 ± 0.41	8.64 ± 0.24	<0.001
MetS [n (%)]	6 (5.13)	21 (75)	<0.001

Abbreviations: HTGW, hypertriglyceridemic waist; SD, standard deviation; BMI, body mass index; WHR, waist to height ratio; BP, blood pressure; SBP, systolic blood pressure; DBP, diastolic blood pressure; Chol, cholesterol; HDL, high-density lipoprotein; LDL, low-density lipoprotein; HOMA-IR, homeostasis model assessment of insulin resistance; ALT, alanine aminotransferase; TyG, triglycerides glucose index; MetS, metabolic syndrome.

**Table 3:** Association between HOMA-IR and the HTGW phenotype.

Independent Variables	OR (95% CI)	*p* Value
HTGW phenotype *Normal* *One* *Two*	reference 4.27 (1.42,12.9) 7.9 (1.94, 32.1)	0.010 0.004
Age (years)	1.29 (0.479, 2.85)	0.033
Sex (boys vs. girls)	1.17 (0.479, 2.85)	0.732
Puberty (No vs. Yes)	1.91(0.579, 6.30)	0.288
HDL (mg/dL)	0.970 (0.935, 1.01)	0.102
BP > 90th (No vs. Yes)	1.03 (0.988, 1.08)	0.156

Abbreviations: HOMA-IR, homeostasis model assessment of insulin resistance; HTGW, hypertriglyceridemic waist; OR, odds ratio; CI, confidence interval; HDL, high-density lipoprotein; BP, blood pressure.

**Table 4:** Association between MetS and HTGW phenotype.

Independent Variables	OR (95% CI)	*p* Value
HTGW phenotype (no vs. yes)	81.4 (13.5, 488.7)	<0.001
Age (years)	1.44 (0.884, 2.362)	0.142
Sex (boys vs. girls)	1.75 (0.293, 10.4)	0.539
Puberty (no vs. yes)	1.11 (0.130, 9.5)	0.923
HDL (mg/dL)	0.872 (0.791, 0.961)	0.006
Total Cholesterol (mg/dL)	1.03 (1.00, 1.06)	0.050

Abbreviations: MetS, metabolic syndrome; HTGW, hypertriglyceridemic waist; OR, odds ratio; CI, confidence interval; HDL, high-density lipoprotein BP, blood pressure.

## Data Availability

The authors confirm that the majority of the data supporting the findings of this study are available within the article. Raw data are available from the authors upon reasonable request. The data are not publicly available due to privacy restrictions.
